# Characterization of the Polysialylation Status in Ovaries of the Salmonid Fish *Coregonus maraena* and the Percid Fish *Sander lucioperca*

**DOI:** 10.3390/cells9112391

**Published:** 2020-10-31

**Authors:** Marzia Tindara Venuto, Joan Martorell-Ribera, Ralf Bochert, Anne Harduin-Lepers, Alexander Rebl, Sebastian Peter Galuska

**Affiliations:** 1 Institute of Reproductive Biology, Leibniz Institute for Farm Animal Biology (FBN), 18196 Dummerstorf, Germany; venuto@fbn-dummerstorf.de; 2 Institute of Genome Biology, Leibniz Institute for Farm Animal Biology (FBN), 18196 Dummerstorf, Germany; martorell-ribera@fbn-dummerstorf.de (J.M.-R.); rebl@fbn-dummerstorf.de (A.R.); 3 Mecklenburg-Vorpommern Research Centre for Agriculture and Fisheries (LFA-MV), 18375 Born, Germany; r.bochert@lfa.mvnet.de; 4 Université de Lille, CNRS, UMR 8576-UGSF-Unité de Glycobiologie Structurale et Fonctionnelle, F-59000 Lille, France; anne.harduin-lepers@univ-lille.fr

**Keywords:** sialic acid, polysialic acid, sialyltransferases, Salmonidae, Percidae, oogenesis, previtellogenesis stages, primordial germ cells, oogonia

## Abstract

In vertebrates, the carbohydrate polymer polysialic acid (polySia) is especially well known for its essential role during neuronal development, regulating the migration and proliferation of neural precursor cells, for instance. Nevertheless, sialic acid polymers seem to be regulatory elements in other physiological systems, such as the reproductive tract. Interestingly, trout fish eggs have polySia, but we know little of its cellular distribution and role during oogenesis. Therefore, we localized α2,8-linked *N*-acetylneuraminic acid polymers in the ovaries of *Coregonus maraena* by immunohistochemistry and found that prevalent clusters of oogonia showed polySia signals on their surfaces. Remarkably, the genome of this salmonid fish contains two *st8sia2* genes and one *st8sia4* gene, that is, three polysialyltransferases. The expression analysis revealed that for *st8sia2-r2*, 60 times more mRNA was present than *st8sia2-r1* and *st8sia4*. To compare polysialylation status regarding various polySiaT configurations, we performed a comparable analysis in *Sander lucioperca*. The genome of this perciform fish contains only one *st8sia2* and no *st8sia4* gene. Here, too, clusters of oogonia showed polysialylated cell surfaces, and we detected high mRNA values for *st8sia2*. These results suggest that in teleosts, polySia is involved in the cellular processes of oogonia during oogenesis.

## 1. Introduction

Sialic acids, a heterogeneous family of acidic monosaccharides, are ubiquitous on the surfaces of eukaryotic cells and are involved in many crucial cellular events [1]. Besides the typical terminal monosialyl residues, sialic acid polymers can also be synthesized in vertebrates. However, in comparison to monosialylation, elongation by further sialic acid residues is mostly restricted to selected cell types and/or distinct points of differentiation [2,3,4]. For instance, proliferating populations of smooth muscle cells are polysialic acid (polySia)-positive during the postnatal development of murine epididymis, whereas at postnatal day 25, no significant polysialylation occurs in these contractile cell clusters [5].

In mammals, sialic acids polymers contain only α2,8-linked *N*-acetylneuraminic acid (Neu5Ac) residues; so far, no polymers with other sialic acids or types of glycosidic bonds (e.g., α2,9-linkages) have been found in vivo. In contrast, fish eggs can contain a high variety of different sialic acid polymers. Inoue and colleagues observed that fertilized and unfertilized eggs from Salmonidae contained sialic acid polymers [6,7,8]. Interestingly, in addition to Neu5Ac homopolymers, they found more α2,8-linked polySia species, such as *N*-glycolylneuraminic acid (Neu5Gc) homopolymers, as well as hybrid structures of different sialic acid species that could also be *O*-acetylated [9,10]. The main protein carrier of these polySia chains seems to be *O*-glycans of the polysialoglycoprotein (PSGP) [8,11].

The sialyltransferases, which catalyze the biosynthesis of α2,8-linked polySia, belong to the α2,8-sialyltransferase (*st8sia*) gene family [12,13]. In mammals, two polysialyltransferases (polySiaTs) are known: ST8SiaII and ST8SiaIV. However, in ray-finned fish, poly-α2,8-sialyltransferase has a particular distribution as a result of several whole-genome duplication (WGD) and gene loss events [14]. For instance, in the salmonid genomes, in addition to *st8sia4*, two *st8sia2* gene loci have been described [15]. Comparatively, in percid genomes, a loss of *st8sia4* took place during evolution, so only one *st8sia2* gene now exists [12,16].

Whereas the presence of polysialyltransferase genes in various fish lineages [16] and the chemical composition of polySia on maturated trout eggs are well known [9,11,17,18,19], only limited knowledge of the occurrence and role of α2,8-linked Neu5Ac polymers during oogenesis is available for fish. For this reason, we analyzed the polysialylation status of the ovaries of maraena whitefish *(Coregonus maraena; C. maraena*), a teleost fish with economic relevance in the Baltic area, which belongs to the salmonid family of bony fishes [20]. The histological distribution of α2,8-linked Neu5Ac polymers in the ovaries of *C. maraena* was compared with the polysialylation status of *Sander lucioperca* (*S. lucioperca*), since the genome of this percid fish contains one *st8sia2* and no *st8sia4* gene, whereas the genome of the salmonid fish *C. maraena* contains two *st8sia2* genes and one *st8sia4* gene [12,16]. Thus, a comparison of their polysialylation statuses is possible regarding different polySiaT configurations.

## 2. Materials and Methods

### 2.1. Sample Collection

Female samples from *C. maraena* were provided by the Institute of Fisheries of the Mecklenburg-Vorpommern Research Center for Agriculture and Fisheries (Born, Germany). Maraena whitefish were raised in a recirculation aquaculture system (RAS), water temperature between 20 and 22 °C, maintained by an automated purification and disinfection system with a 16:8-h day-night cycle. From August to November, water temperature was reduced during 74 days to 4 °C and fish were kept at that temperature for out-of-season reproduction. Day-night light cycles were adapted during that period according to natural conditions. Fish were fed with commercial pellets feed. The fish were caught monthly using a sieve with a net. Specimens were sacrificed following the standards described in the German Regulations for Animal Protection (2006) (TierSChG) and current German Regulations for Animal Protection and Slaughter as of 20 December 2012 (TierSchlV). Ovarian tissues from each fish were used for paraffin histology, and the remaining tissue was snap frozen at −80 °C for future protein extraction and RNA isolation.

Ovarian *S. lucioperca* samples, used for immunohistochemistry, were provided by Fischerei Loch (Hohen Sprenz, Germany). The ovaries were collected from animals processed for food production. The animals are from cage systems, which are placed in the lake Hohen Sprenz.

For mRNA analysis, *S. lucioperca* were provided by the Institute for Fishery of the State Research Center for Agriculture and Fisheries Mecklenburg-Western Pomerania (Hohen Wangelin, Germany) and maintained in the RAS facilities of the Leibniz institute for farm animal biology (FBN). The temperature of the water was set at 22 °C, with a 12:12-h day-night cycle. Water quality was regularly monitored and maintained by automated purification and disinfection (bio-filter and UV light).

### 2.2. Western Blotting

Proteins from the ovaries of *C. maraena* were extracted with a TriPrep kit following the manufacturer’s instructions (Nucleospin, Macherey-Nagel, Düren, Germany). The resulting samples were resolved in 1 × RIPA Buffer, and one aliquot of each sample was treated with endoneuraminidase (endoN) (6.7 µg/mL) for 1 h at 37 °C to degrade α2,8-linked Neu5Ac polymers. In addition, a color prestained protein standard was used (broad range 10–250 kDa; Cell signaling). The samples were subjected to 7% SDS/PAGE and subsequently transferred to a PVDF membrane. The membrane was blocked with 0.5% no-fat dry milk in TBS 1× buffer. Immunostaining against polySia was done with monoclonal antibody (mAb) 735 (1 µg/mL) in 5% bovine serum albumin (BSA), which recognizes α2,8-linked Neu5Ac polymers with a degree of polymerization (DP) ≥ 8. Martina Mühlenhoff (MHH Hannover, Germany) provided mAb 735 and endoN [21,22]. Horseradish peroxidase (HRP)-conjugated secondary antibodies (donkey anti-mouse, Dianova, Hamburg, Germany) were applied for visualization by chemiluminescence signal using ECL Prime. Subsequently, a Coomassie staining of the PVDF membrane was performed (Coomassie Brilliant Blue R Dye, Merck-Millipore, Darmstadt, Germany) (staining solution: 0.1% (*w/v*) Coomassie blue in 50% (*v/v*) methanol, 7% (*v/v*) acetic acid) to control the protein transfer (loading control), since Coomassie staining is compatible with immunoblotting [23]. Pictures of the chemiluminescence signal and the Coomassie staining were taken with a Bio-Rad imaging system (ChemiDoc^TM^ MP, Feldkirchen, Germany).

### 2.3. Real-Time Quantitative PCR (RT-qPCR)

The total RNA from *C. maraena* ovaries was purified with the TriPrep kit (Nucleospin, Macherey-Nagel, Düren, Germany) according to the manufacturer’s protocol. *S. lucioperca* ovaries were homogenized using 2.8 mm ceramic beads (Precellys, VWR/Avantor) at 6000 rpm for 30 s using the Precellys 24 Homogenizer (VWR/Avantor). The whole RNA was extracted using TRIzol Reagent (Life Technologies, Carlsbad, CA, USA) and subsequently purified with the RNeasy Mini Kit (Qiagen, Hilden, Germany). The quantity of RNA was measured with a NanoDrop One^c^ photomometer (Thermo Fisher Scientific, Darmstadt, Germany). In addition, the RNA integrity was verified by electrophoresis on 1%-agarose gels. RNA was reverse transcribed to cDNA using the SensiFAST cDNA Synthesis Kit (Bioline, London, UK) following the manufacturer’s protocols.

Real-time quantitative PCR (RT-qPCR) was used to determine the expression of the polySiaTs: the two duplicated genes *st8sia2 r-1*, *st8sia2 r-2,* and *st8sia4* for *C. maraena* and *st8sia2* for *S. lucioperca*. For this purpose, the gene-specific primers were designed using software (Pyrosequencing Assay Design software v.1.0.6; Biotage, Uppsala, Sweden) to amplify the fragments between 121 and 226 bp. To identify the *st8sia2* sequences in maraena whitefish, the orthologous sequences from rainbow trout *Oncorhynchus mykiss* (*O. mykiss*) were blasted against the transcriptome of maraena whitefish [20], deposited at the NCBI Sequence Read Archive (accession code: SRP066290; BioProject PRJNA302355).

The following primers were used for *C. maraena*:

*st8sia2* *r-1:* 5´AGCCTCATCAGGAAGAACATCC-3´ (sense)
  5´-TTCCCTACGATGGCACAGCGT-3´ (antisense)
*st8sia2* *r-2:* 5´-CGTTCAACAGGAGCCTCTCTAA-3´ (sense)
  5´-TTCCCTACGATGGCACAGCGC-3´ (antisense)
*st8sia4:*       5´-ATGATAAGGAAGGACGTGCTGC-3´ (sense)
       5´-TGTTGAGCGTTCGGCGTCTGT-3´ (antisense)

The *st8sia2* sequences in pikeperch were obtained from the recently published *S. lucioperca* genome [24]. For *st8sia2* in *S. lucioperca*, the following primers were designed and applied:

*st8sia2:*       5´-GAGGAAGAAACTGCAAATACTGG-3´ (sense)
       5´-AGTTGTTTGACGAGAGCTTGACA-3´ (antisense)

RT-qPCR was run with the LightCycler 96 System (Roche, Mannheim, Germany) in a 12-µL reaction volume containing 16.6 ng/5 µL cDNA for *C. maraena* and 50 ng/5 µL cDNA for *S. lucioperca*, 1 µL of sense and antisense primers each, and 6 µL of SensiFast SYBR No-ROX Mix (Bioline). The RT-qPCR program comprised a denaturation phase (95 °C, 5 min) followed by 40 cycles of denaturation (95 °C, 5 s), annealing (60 °C, 15 s), elongation (72 °C, 15 s), and fluorescence measurement (72 °C, 10 s). PCR products were separated on 3% agarose gels and documented by the iBright FL1000 imaging system (Invitrogen/Thermo Fisher Scientific, Darmstadt, Germany). In addition, the specific melting curve of each target gene was evaluated to check for unspecific products. The obtained real-time data were normalized against a factor based on the geometric mean values from three suitable reference genes encoding the eukaryotic elongation factor 1-alpha 1 (gene *eef1a1*) and two ribosomal protein units (genes *rpl9* and *rpl32* for *C. maraena* or *rpl32 and rps5* for *S. lucioperca*) [25].

### 2.4. Histological Sample Preparation

For histology, tissue samples were cooled on ice and fixed in Bouin (picric acid, formaldehyde, and acetic acid, 15/5/1, *v/v/v*) for 24 h. In parallel, samples were fixed for 48 h using a Bouin solution with 2% sucrose. Thereafter, the samples were successively dehydrated with a stepwise alcohol gradient and cleared in xylene followed by isopropanol for 27 h in an automatic tissue processor (MT, SLEE, Mainz, Germany). All the samples were embedded after dehydration in a paraffin station (MPS/P2, SLEE, Mainz, Germany). Paraffin sections (5 µm) were cut with a microtome (LEICA RM 2165, Wetzlar, Germany) and transferred to glass slides. After deparaffinization (1 h at 60 °C, rehydrated in different alcohol steps and pressure-cooked in Tris buffer pH 9 for 3 min), the tissues were stained with hematoxylin and eosin (HE). The stained tissue sections were examined with a laser scanning microscope (Carl Zeiss Axio Observer.Z1, Oberkochen, Germany) 

### 2.5. Immunohistochemistry Procedure

For immunohistochemistry, the deparaffinized sections were washed with PBS containing 0.2% (*w/v*) immunoglobulin G (IgG)-free BSA (Carl Roth, Karlsruhe, Germany). After three washes, tissue sections were blocked with PBS containing 2% BSA (*w/v*) for 1 h at 37 °C. To immunolocalize α2,8-linked polySia, mAb 735 (0.4 µg/mL) was used. To confirm the specific binding of mAb 735, we used two control strategies: (1) omitting the first antibody and (2) degrading the α2,8-linked polySia with endoN overnight at 37 °C (1.34 µg/mL). The tissue sections of both controls were on the same slide as the normally stained sample.

The incubation with the primary antibody was prepared overnight in PBS containing 0.2% (*w/v*) IgG-free BSA at 4 °C. Subsequently, the tissue sections were washed and incubated for 1 h at room temperature (RT) with a secondary antibody (envision kit+ system-HRP labeled polymer anti-mouse; Dako, Jena, Germany). After washing, the sections were revealed using a peroxidase chromogen for immunohistochemistry (IHC) SIGMAFAST 3,3’-Diaminobenzidine (DAB)-tab (Sigma-Aldrich, St. Louis, MO, USA). The nuclei were stained with hematoxylin for 10 s.

### 2.6. Fluorescent Staining

For the visualization of polySia, instead of the HRP-conjugated secondary antibody, the samples were incubated for 1 h at RT with Alexa Fluor 488-conjugated (Fab)_2_ fragment of goat anti mouse IgG (H + L) (A11017 Carlsbad, California, CA, USA) (10 µg/mL in 1% BSA) or with Alexa Fluor 568-conjugated goat anti-mouse IgG (H + L) secondary antibody (A11031 Abcam, Cambridge, UK) (1 µg/mL in 1% BSA). For a parallel staining of DDX4, also known as VASA protein, the tissue sections were blocked with PBS containing 5% BSA and goat serum for 1 h at 37 °C. Thereafter, a rabbit IgG polyclonal antibody against DDX4 (ab 13840 Abcam, Cambridge, UK) (1 µg/mL in 1% BSA) was used (overnight at 4 °C). The antibodies bound against DDX4 were labeled with an Alexa Fluor 647-conjugated goat anti-rabbit IgG (H + L) secondary antibody (A212245 Life Technologies, Carlsbad, California) (1 µg/mL in 1% BSA) for 1 h at RT. The DNA was stained with 4′,6-diamidino-2-phenylindole (DAPI) for 10 min at RT. Finally, a fixation step was performed using 2% paraformaldehyde (PFA) for 20 min at RT. The stained tissue sections were examined with a laser scanning fluorescence microscope (Carl Zeiss Axio Observer.A1, Oberkochen, Germany).

### 2.7. Synteny Analysis

For the synteny (blocks of orthologous genes) and paralogy analyses, the *st8sia* gene loci and their neighbor genes were localized using NCBI BLAST search, and the detection of paralogous genes was assessed by manual chromosome walking. We chose genes physically close to the gene of interest to identify only the syntenic segment of interest.

### 2.8. Statistical Analysis

The calculated values were analyzed with Graph Pad Prism 8.4.3 software (GraphPad Software, San Diego, CA, USA) using a Mann–Whitney test. The differences were considered statistically significant at *p* < 0.05.

## 3. Results

### 3.1. Polysialylation in Ovary of C. maraena

**Figure 1:**
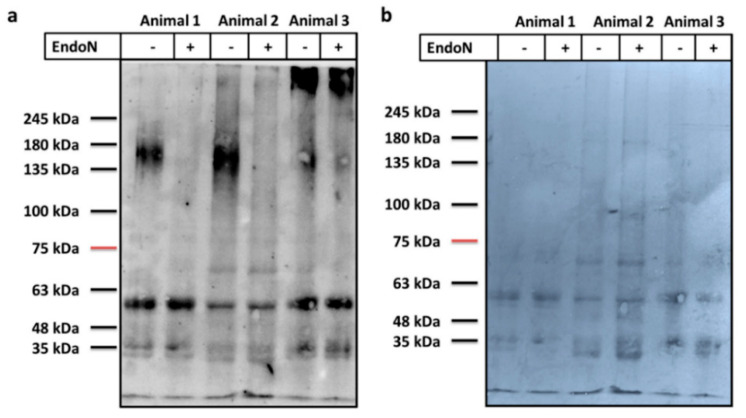
Polysialylation status in maraena whitefish ovary. Proteins (5 µg per lane) were separated by 7% SDS-PAGE, and (**a**) polySia was visualized by Western blotting. An aliquot of each sample was treated with endoN to degrade polySia. The polysialylation status of three independent ovaries (three different animals) is displayed, and molecular masses (kDa) of standard proteins are indicated. (**b**) For loading control, the membrane was stained with Coomassie blue.

Kitajima and colleagues found that α2,8-linked polyNeu5Ac was present on salmonid fish eggs [9] and that all polySiaTs were expressed in rainbow trout ovaries [15]. Based on their studies, we investigated the ovaries of *C. maraena*, which also belongs to the salmonid lineage, for α2,8-linked Neu5Ac polymers with a DP ≥ 8 using mAb 735 [21,26]. To this end, ovarian tissue was lysed, and the protein was enriched. After protein separation by SDS-PAGE and Western blotting against polySia, broad signals between 135 and 180 kDa were visualized, which were absent or reduced after endoN treatment (Figure 1). EndoN degrades α2,8-linked Neu5Ac polymers [22,27]. The extensive smear is typical for polysialylated proteins and might result from a heterogeneous polySia chain length distribution on polySia carriers [28,29]. In the case of animal 3, the signal was not completely abolished. This could result from an incomplete digest or a background signal. The visualized protein bands, which showed comparable signal intensities in the endoN-treated and untreated aliquots (low kDa area and in animal 3 proteins > 245 kDa) seem to be the result of an unspecific binding of the applied antibodies. In sum, the obtained results strongly suggest that polySia is present in the ovaries of *C. maraena*. 

It should, however, be noted that it cannot be unambiguously denied that the mAb 735 might additionally visualize other polySia species than α2,8-linked Neu5Ac homopolymers. It has been suggested that anti-polySiaNeu5Ac antibodies (e.g., mAb 12E3) may also bind heteropolymers of Neu5Ac and Neu5Gc residues, even if the binding efficiency is lower [30,31]. Furthermore, endoN can also degrade Neu5G-containing polySia in addition to Neu5Ac homopolymers but with lower efficiency [30,31].

### 3.2. Expression Levels of PolySiaT Genes in C. maraena Ovaries

**Figure 2:**
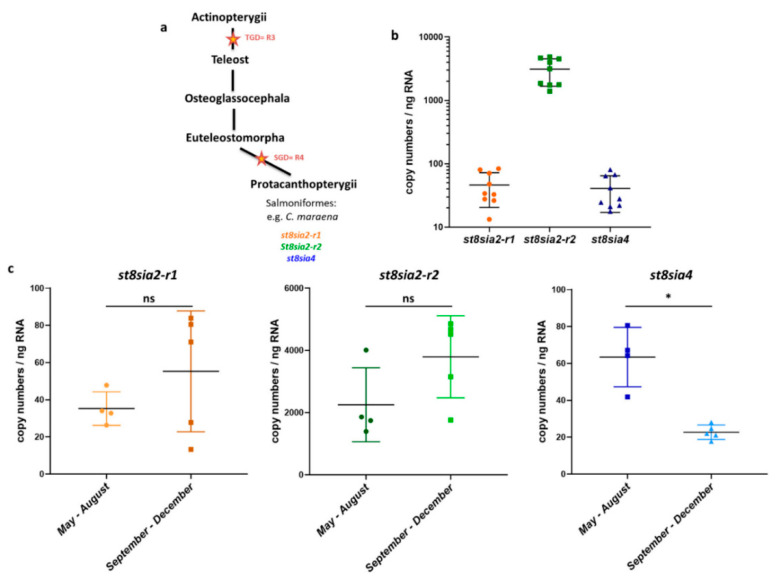
Expression profiling of polysialyltransferase genes in *C. maraena* ovaries. (**a**) Overview of the polyST family evolution in the Protacanthopterygii. Orange stars correspond to the whole-genome duplications (WGDs): R3 (teleost-specific duplication, TGD) and R4 (salmonid-specific duplication, SGD). For a more comprehensive illustration, please see Supplemental Figure S1. (**b**) The transcript levels of *st8sia2-r1* (orange), *st8sia2-r2* (green), and *st8sia4* (blue) were determined in ovaries from maraena whitefish (n = 9 animals). Data are plotted on a logarithmic scale. (**c**) The transcript levels of the polysialyltransferase genes during spring and summer (n = 4 animals) and autumn (n = 5 animals) are separately displayed. All error bars represent the standard deviation. Non-significant, ns; * *p* < 0.05.

Following the polySia-positive Western blot results, the expression levels of the polySiaT genes were examined using qPCR. In rainbow trout ovaries, three distinct genes are expressed: two *st8sia2* variants and one *st8sia4* gene [15]. A recent phylogenetic analysis suggests that the two *st8sia2* genes resulted from a fourth duplication of the whole genome in Salmonidae (SGD = R4) after the teleost radiation (TGD = R3) [16] (Figure 2a and Supplemental Figure S1). Thus, the genome of maraena whitefish encodes three polySiaTs, just as in rainbow trout.

To determine the relative transcript levels of the polysialyltransferase genes in the ovaries of *C. maraena*, specific primer pairs for *st8sia2-r1*, *st8sia2-r2*, and *st8sia4* were applied. The qPCR analysis revealed that the numbers of *st8sia2-r1* transcripts (about 46 copies/ng RNA) and *st8sia4* transcripts (about 40 copies/ng RNA) were comparably low (Figure 2b). Remarkably, the number of *st8sia2-r2* transcripts was more than 60 times higher (about 3110 copies/ng RNA).

However, it must be mentioned that the ovaries of the nine whitefish were collected from May until December. In most teleost species, reproduction is cyclic, and seasonality attends several physiological alterations [32]. Concerning the polySiaTs, season-dependent expression is possible in rainbow trout ovaries, since in September, the mRNA level of *stx-ov* (in *C. maraena st8sia2-r1*) increase and the transcripts of *st8sia4* tend to decrease [15]. For this reason, we separately summarized the determined mRNA levels of the *C. maraena* samples, which were collected during “summer” (from May until August) and “autumn” (from September until December). The mRNA levels for both ST8SiaII enzymes of *C. maraena* slightly increased, although the changes were not statistically significant (Figure 2c). In contrast, the *st8sia4* transcripts slightly decreased. 

In sum, the results demonstrate that ST8SiaII-r2 is the dominantly expressed polysialyltransferase in *C. maraena* ovaries.

### 3.3. Localization of Polysia in Ovaries of Maraena Whitefish

**Figure 3:**
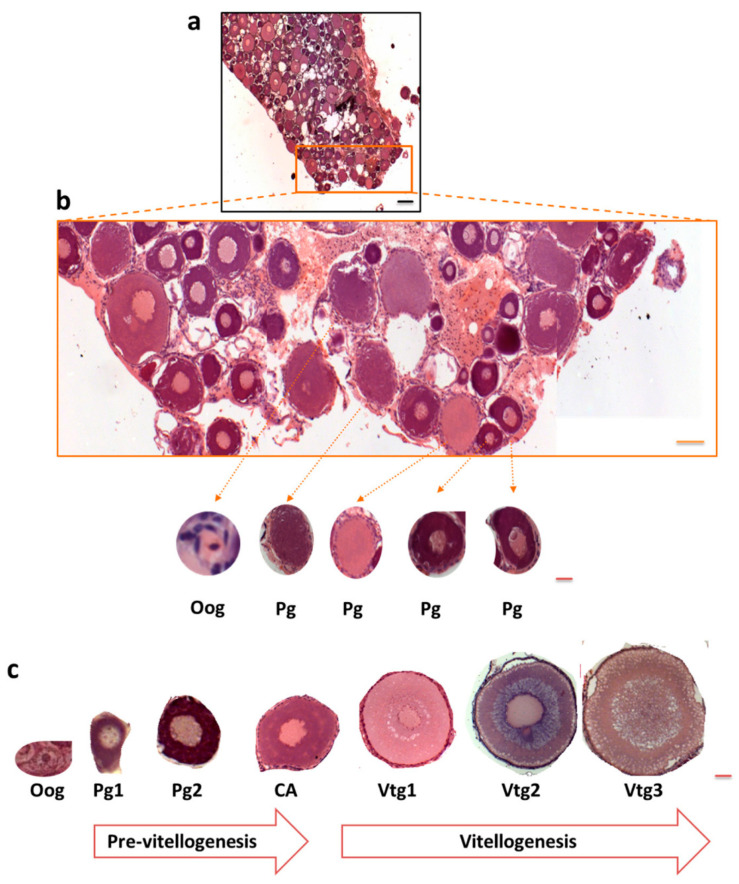
Histological section of *C. maraena* ovary during previtellogenesis. The ovarian tissue was stained with HE; (**a**) black scale bar 200 µm, (**b**) orange scale bar 100 µm, (**b**,**c**) red scale bar 10 µm. The colored box approximately indicates the enlarged areas. (**b**,**c**) Previtellogenesis and vitellogenesis stages in *C. maraena* ovary. The following cells are displayed: oogonia (Oog); oocytes in primordial growth (Pg) and early and late primary growth (Pg1, Pg2, respectively); cortical alveoli (CA); primary, secondary, and tertiary vitellogenesis (Vtg1, Vtg2, and Vtg3, respectively). This schematic presentation of oogenesis is based on [36,37].

Oogenesis in fish is a dynamic process [33]. Remarkably, oogonia can proliferate throughout the lifetime of fish, and mitosis can take place in mature ovaries, whereas in mammals, its proliferation is restricted to the embryonic and fetal periods [34,35]. Maraena whitefish have asynchronous gonad development, with different cell types being frequently present in close proximity. Figure 3 shows an example of this non-homogeneous ovarial stage organization.

**Figure 4:**
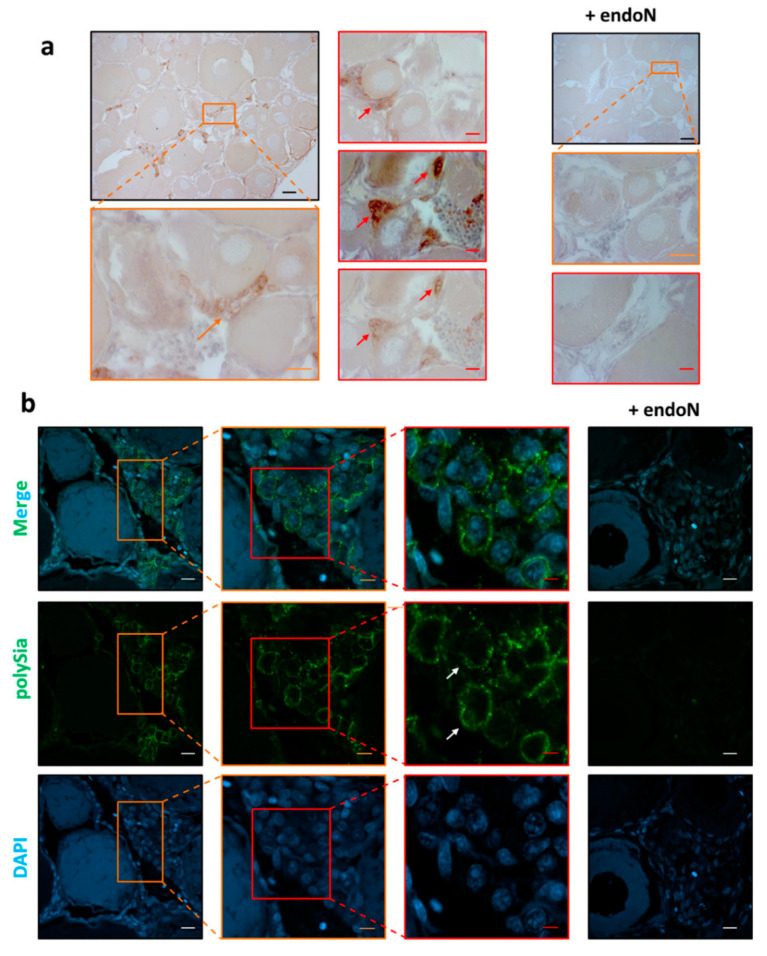
Histological analysis of the polysialylation status in ovaries from *C. maraena*. mAb 735 was applied to detect polySia. (**a**) For immunohistochemical visualization, an HRP-conjugated secondary antibody was used. For negative control, polySia was degraded by endoN. Nuclei were stained with hematoxylin. Selected polySia-positive cells are indicated with orange and red arrows. Black scale bars: 100 µm; orange scale bars: 20 µm; red scale bars: 10 µm. For polySia staining and the respective endoN-digest on serial sections, please see Supplemental Figure S2a. (**b**) PolySia staining was performed with a combination of mAb 735 against polySia and an Alexa Fluor 488-conjugated secondary antibody (green). Controls were performed by depolysialylating with endoN (+ endoN). Nuclei were stained with DAPI (blue). The extensive DAPI staining in higher stages of egg development is common [38]. Selected polySia-positive cells are labeled with white arrows showing, in contrast to higher stages of egg development, a typical nuclear staining. White scale bars: 20 µm; orange scale bars: 10 µm; red scale bars 5 µm. The colored boxes approximately indicate the enlarged areas.

To investigate the localization of polySia in ovaries, tissue sections were treated with mAb 735 followed by differently conjugated secondary antibodies (HRP, Figure 4a; Alexa Fluor 488, Figure 4b). The obtained results revealed that polySia-positive cell clusters are mainly localized between cell units in previtellogenic stages (Figure 4). The staining did not take place when polySia was degraded with endoN.

**Figure 5:**
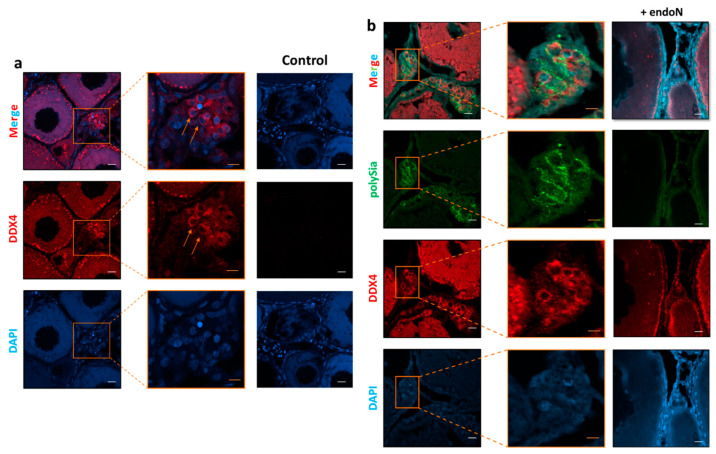
Localization of oogonia and polySia in *C. maraena* ovary. (**a**) DDX4 staining was performed with a DDX4 antibody and an Alexa Fluor 647-conjugated secondary antibody (red). Selected oogonia are indicated with orange arrows. Controls were performed by omitting the first antibody (control). (**b**) PolySia was stained in parallel using a combination of mAb 735 against polySia and an Alexa Fluor 488-conjugated secondary antibody (green). For negative control, polySia was degraded by endoN. Nuclei were stained with DAPI (blue). White scale bars: 20 µm; orange scale bars: 10 µm. The colored boxes approximately indicate the enlarged areas.

Interestingly, Nakamura et al. found that oogonia in *Oryzia latipes* ovaries formed clusters of proliferating cells between cells in previtellogenic stages, which can be stained with antibodies against DEAD-box helicase proteins [35]. Based on these studies, we used polyclonal antibodies against DEAD-box helicase 4 (DDX4). In parallel, polySia was visualized. The results demonstrate that several DDX4-positive cells are polySia positive (Figure 5) and suggest that oogonia nests might be a source of polySia in the analyzed ovaries of maraena whitefish.

The heterogeneous distribution of polySia-positive cell clusters in the tissue sections explains the high level of variance in the polyST expression levels (Figure 2) and that the intensity of polySia Western blot signals (Figure 1) are not identical in all animals. The tissue parts of ovaries will generally have a different composition of polySia-positive and -negative cells.

### 3.4. Polysialylation in Ovaries of S. lucioperca

**Figure 6:**
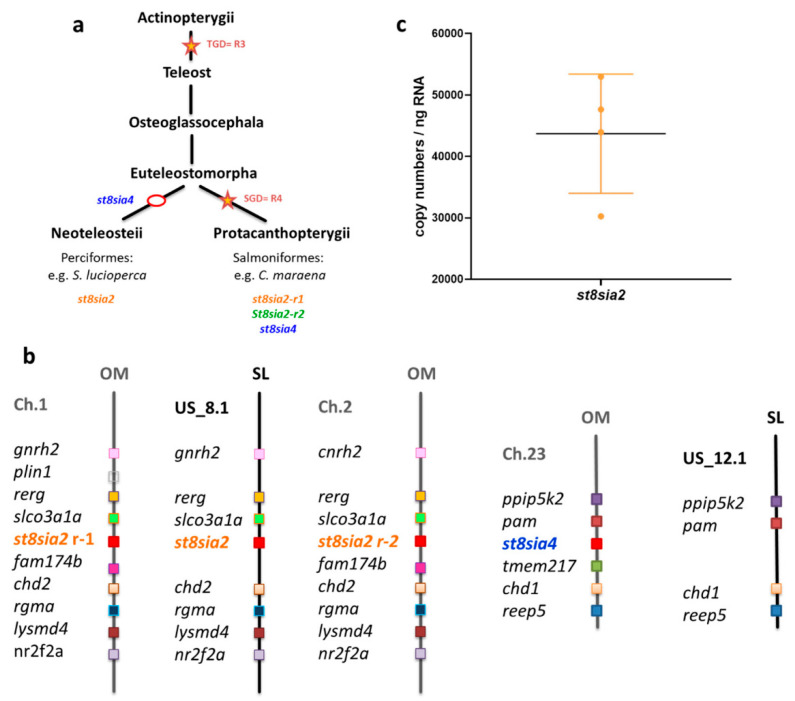
Expression level of *st8sia2* in ovaries from *S. lucioperca*. (**a**) Overview of the polyST family evolution in Actinopterygii. Orange stars correspond to the WGDs, and red circles indicate the loss of *st8sia4.* R3 (teleost-specific duplication, TGD) and R4 (salmonid-specific duplication, SGD). For a more comprehensive illustration, please see Supplemental Figure S1. (**b**) Schematic representation of the chromosomal location of *st8sia* genes, syntenic relationships of polySiaTs, and the neighboring gene loci retrieved from rainbow trout (*O. mykiss*, OM) and pike perch (*S. lucioperca*, SL). The orthologues were determined using information from ENSEMBL and NCBI. (**c**) The transcript level of *st8sia2* was determined in *S. lucioperca* ovaries (*n* = 4 animals).

In *C. maraena* ovaries, with ST8SiaII-r2, only one of the three polySTs (ST8SiaII-r1, ST8SiaII-r2, and ST8SiaIV) was expressed with copy numbers higher than 1000 (Figure 2b). The other two, ST8SiaII-r1 and ST8SiaIV, seemed present at significantly lower levels (less than 100 copy numbers per ng RNA). Thus ST8SiaII-r2 may synthesize the most polySia chains, which are present in clusters of oogonia in *C. maraena*. However, it is possible that ST8SiaII and IV are cooperatively involved in polysialylation, as hypothesized for rainbow trout ovaries [15]. Interestingly, in other teleost lineages, such as Neoteleostei, ST8SiaIV was lost and only one variant of ST8SiaII is present (Figure 6a and Supplemental Figure S1). Consequently, the availability of various polySiaTs is restricted to ST8siaII.

We conducted synteny and paralogy analyses of the *st8sia2-r1* and *st8sia2-r2* loci in genomes of Salmonidae. Figure 6c illustrates the presence of a paralogon including these two gene loci and *gnrh*, *rerg*, *slco3a1a*, *fam174b*, *chd2*, *rgma*, *lysmd4*, and *nr2f2a* paralogues on chromosomes 1 and 2 in the rainbow trout *O. mykiss*. This series of genes is well conserved in the pike perch *S. lucioperca* on scaffold (NW_022173278.1), further suggesting that the *C. maraena st8sia2-r1* and *st8sia2-r2* are co-orthologues of the *S. lucioperca st8sia2* and could have the same function in fish ovaries.

In *S. lucioperca* ovary samples, the Q-PCR analysis of the *st8sia2* expression levels revealed that it is more than 10 times higher (about 48,133 copies/ng RNA) than *st8sia2-r2*, the dominant polysialyltransferase in maraena whitefish ovaries (Figure 6c). This might be a compensatory effect of the loss of ST8SiaIV and the absence of a second ST8SiaII polysialyltransferase. However, it must be mentioned that the environmental/breeding conditions of *C. maraena* were different compared to *S. lucioperca.* Thus, a quantitative comparison is hardly possible. Nevertheless, it is obvious that, also in *S. lucioperca* ovaries, polysialyltransferases are expressed.

**Figure 7:**
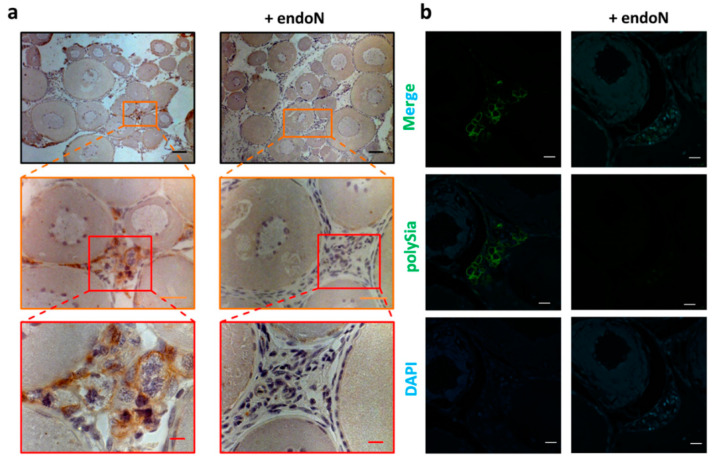
Localization of polySia in ovaries from *S. lucioperca*. mAb 735 was applied to detect polySia in *S. lucioperca* ovary tissue in the previtellogenesis stage. For negative control, polySia was degraded by endoN. (**a**) For immunohistochemical visualization, an HRP-conjugated secondary antibody was used. Nuclei were stained with hematoxylin. Black scale bar: 100 µm; orange scale bar: 20 µm; red scale bar: 10 µm. For polySia staining and the respective endoN-digest on serial sections, please see Supplemental Figure S2b. (**b**) PolySia staining was performed with a combination of mAb 735 and an Alexa Fluor 488-conjugated secondary antibody (green). Nuclei were stained with DAPI (blue). White scale bars: 20 µm. The colored boxes approximately indicate the enlarged areas.

**Figure 8:**
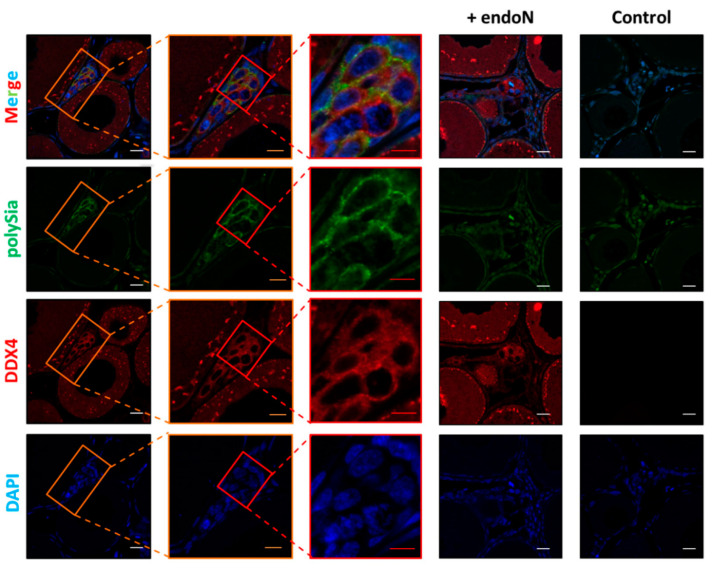
Localization of DDX4 and polySia in *S. lucioperca* ovary. PolySia staining was performed with a combination of mAb 735 against polySia and an Alexa Fluor 568-conjugated secondary antibody (green). For negative control, polySia was degraded by endoN. Germ cells were visualized with a polyclonal antibody against DDX4 in combination with an Alexa Fluor 647-conjugated secondary antibody (red). Controls were performed by omitting the first antibodies (control). Nuclei were stained with DAPI (blue). White scale bars: 20 µm; orange scale bars: 10 µm; red scale bars 6 µm. The colored boxes approximately indicate the enlarged areas.

Subsequently, the polysialylation status of *S. lucioperca* ovaries was also investigated by immunohistochemistry. Consistent with the results from *C. maraena*, in *S. lucioperca*, clustered cell populations were polySia positive (Figure 7). Ovarian tissue sections from these organisms were also stained against polySia and DDX4 in parallel. As illustrated in Figure 8, several oogonia also displayed a co-staining of polySia and DDX4 in *S. lucioperca*. Thus, despite the absence of ST8SiaIV, polySia is synthesized in these cell populations, likely by the ST8SiaII enzyme. 

The heterogeneity of polySia-positive and -negative cells in a cell population is common and depends very often on the state of activation [2,3,4,39]. In the case of neuronal cells, for instance, polySia supports the migration of the cells [40] or influences access to growth factors [41]. Since in the ovaries numerous remodeling processes take place, comparable polySia-influenced mechanisms are conceivable. However, the obtained results do not enable valid conclusions to be drawn.

## 4. Conclusions

A special characteristic of dynamic oogenesis in fish is the possibility of oogonia proliferating throughout the fish lifetime [31]. Intriguingly, our data revealed that in Salmonidae and Percidae, clusters of these cells exhibited polySia on their cell surfaces, although the fish families had different setups of polySiaTs. Whereas the Salmonidae genome contained three genes for polySiaTs, namely *st8sia2-r1*, *st8sia2-r2*, and *st8sia4*, Percidae could only use *st8sia2*. Interestingly, the *st8sia2* gene in Percidae was flanked by genes comparable to *st8sia2-r1 and st8sia2-r2* in Salmonidae. In line with this observation, in *C. maraena*, the mRNA levels of *st8sia2-r2* were 60 times higher than the values for *st8sia2-r1* and *st8sia4*. Thus, it seems that polysialylation of oogonia is driven by ST8SiaII variants in both fish families and that polySia is involved in the cellular processes of germ cells during oogenesis in fish. Altogether, these observations further suggest a conserved function for polysialylation found in fish oogonia. Since it is well known that polySia is essentially involved in migration and proliferation processes of neural precursor cells [39], a comparable function in oogonia seems to be likely.

## Supplementary Materials

The following are available online at https://www.mdpi.com/2073-4409/9/11/2391/s1, Figure S1: Scenario illustrating the evolutionary history of the polysialyltransferase genes in Salmoniformes and in Perciformes; https://www.mdpi.com/2073-4409/9/11/2391/s2, Figure S2: Histological analysis of the polysialylation status in ovaries from *C. maraena* and *S. lucioperca*.
