# Supplementary material for: Characterization of the Polysialylation Status in Ovaries of the Salmonid Fish Coregonus maraena and the Percid Fish Sander lucioperca
Source: Cells. 2020 Oct 31;9(11):2391. doi: 10.3390/cells9112391 (PMC7693511; doi:10.3390/cells9112391)
Supplement: Supplementary file 1 [file cells-09-02391-s001.zip › Supplemental figure 1.pdf]

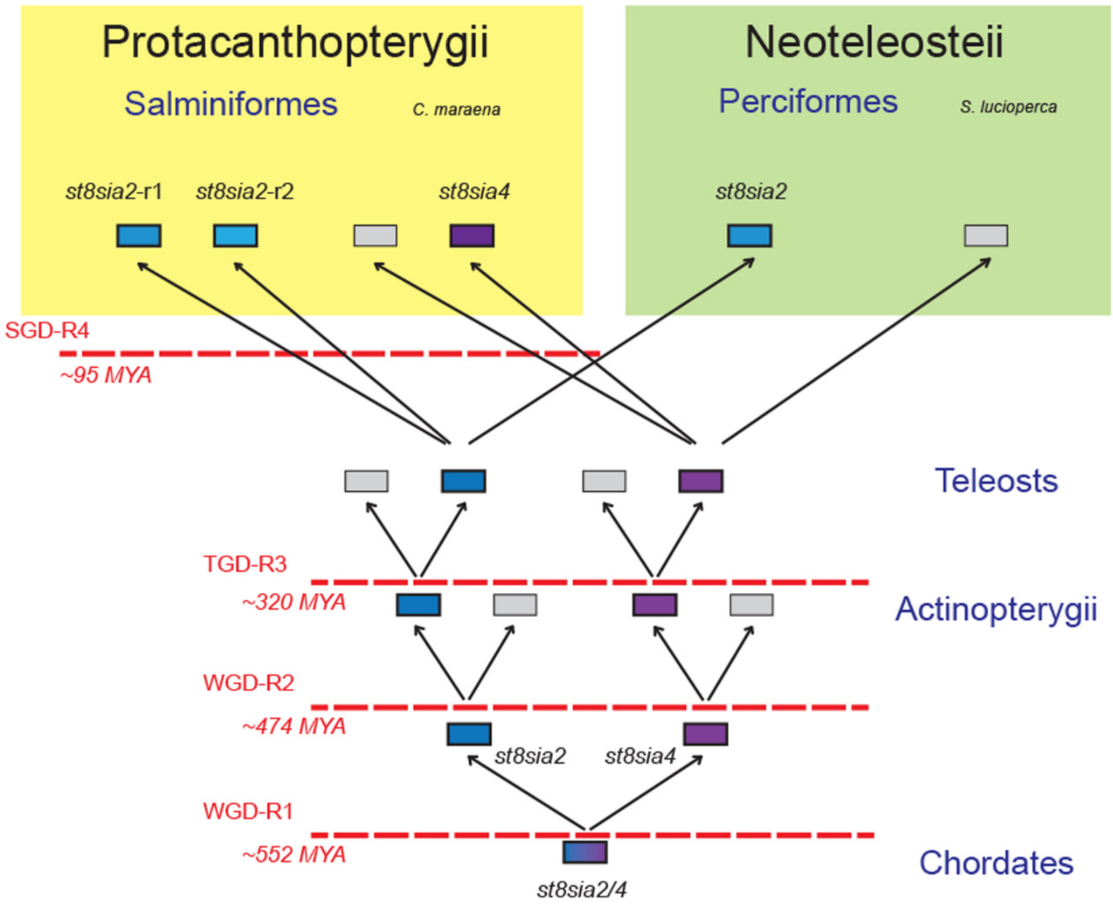

Supplemental figure 1: Scenario illustrating the evolutionary history of the polysialyltransferase genes in Salmoniformes and in Perciformes. For each gene locus, the duplication events are localized relatively to the two rounds of WGD-2R that occurred respectively around 552 and 474 million years ago (MYA) at the base of vertebrates' evolution (WGD-R1 and WGD-R2), the one specific to teleost fishes (TGD-R3) 320 MYA, and the one specific to Salmonidae (SGD-R4) that occurred about 95 MYA. The presence (full colored boxes) or loss (full grey boxes) of *st8sia2* and *st8sia4* genes is indicated in chordates, in Actinopterygii, in teleosts, in Salmoniformes like *Coregonus maraena* (on the left side) and in Perciformes like *Sander lucioperca* (on the right side).
