# Supplementary material for: Characterization of the Polysialylation Status in Ovaries of the Salmonid Fish Coregonus maraena and the Percid Fish Sander lucioperca
Source: Cells. 2020 Oct 31;9(11):2391. doi: 10.3390/cells9112391 (PMC7693511; doi:10.3390/cells9112391)
Supplement: Supplementary file 1 [file cells-09-02391-s001.zip › Supplemental figure 2.pdf]

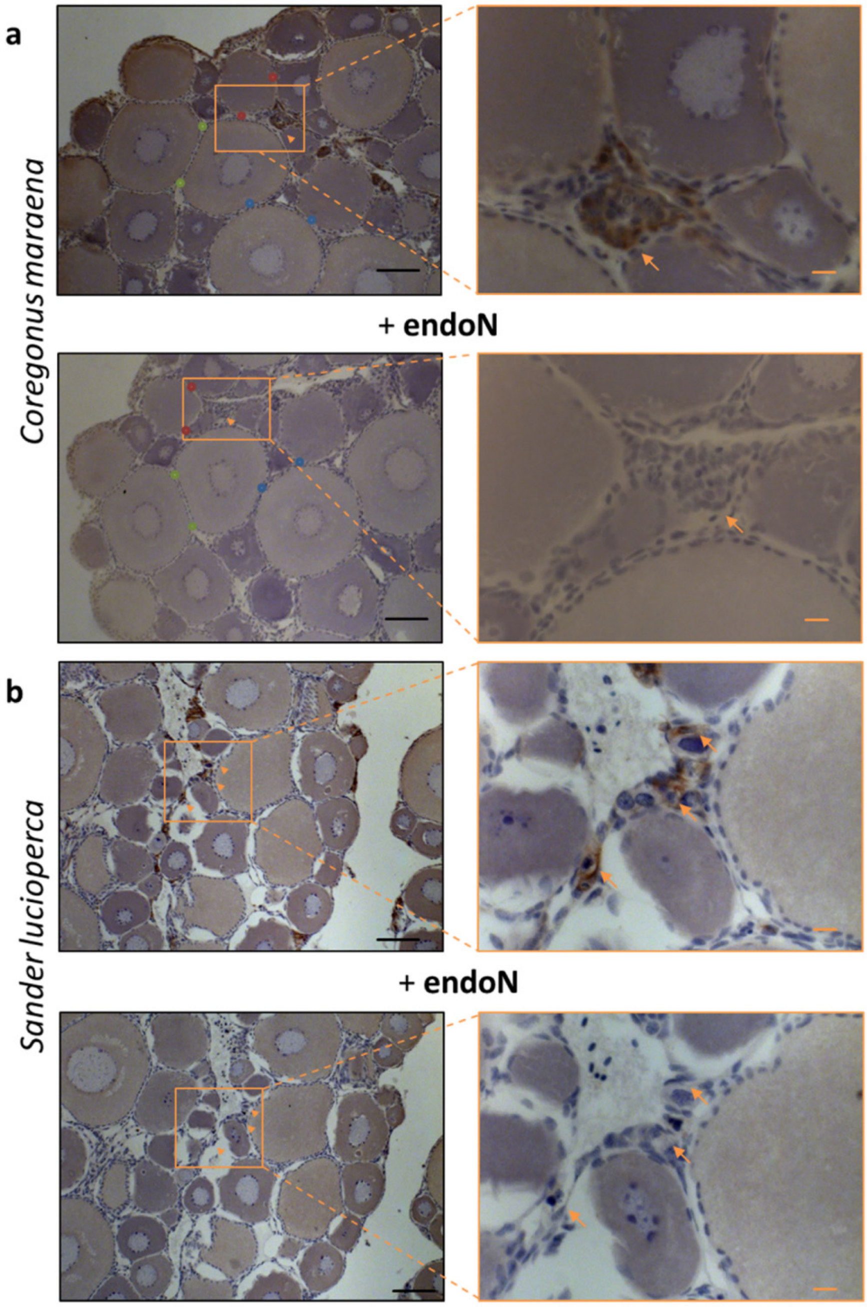

Supplemental figure 2: Histological analysis of the polysialylation status in ovaries from (a) *C. maraena* and (b) *S. lucioperca*. To detect polySia, mAb 735 was applied using 3 µm tissue sections. For immunohistochemical visualization, an HRP-conjugated secondary antibody was applied. For negative control, polySia was degraded by endoN on the following serial section. Nuclei were stained with hematoxylin. Selected polySia-positive cells are indicated with orange arrows. The colored spots should help to find characteristic areas. Black scale bars: 100 µm; orange scale bars: 10 µm. The colored boxes approximately indicates the enlarged areas.
